# Early restrictive fluid balance is associated with lower hospital mortality independent of acute disease severity in critically ill patients on CRRT

**DOI:** 10.1038/s41598-021-97888-y

**Published:** 2021-09-14

**Authors:** Panu Uusalo, Tapio Hellman, Eliisa Löyttyniemi, Julia Peltoniemi, Mikko J. Järvisalo

**Affiliations:** 1 Department of Anaesthesiology and Intensive Care, University of Turku, Kiinamyllynkatu 4-8, P.O. Box 51, 20521 Turku, Finland; 2 Perioperative Services, Intensive Care and Pain Medicine, Turku University Hospital, Turku, Finland; 3 Kidney Center, Turku University Hospital, Turku, Finland; 4 Department of Biostatistics, University of Turku, Turku, Finland

**Keywords:** Continuous renal replacement therapy, Acute kidney injury

## Abstract

Fluid overload (FO) with coincident acute kidney injury has been associated with increased mortality. However, it is unclear whether FO is an independent determinant of mortality for disease severity. We aimed to explore whether the development of fluid balance (FB) during the first 72 h of continuous renal replacement therapy (CRRT) is independently associated with hospital mortality. All patients admitted to a single centre ICU requiring CRRT for at least 24 h between years 2010–2019 were included. Extracted data included patient demographics and clinical parameters including daily cumulative fluid balance (FB^cum^), lactate, SOFA score and vasoactive requirement at the initiation and during the first 72 h of CRRT. 399 patients were included in the analysis. Hospital survivors had a significantly lower FB^cum^ at CRRT initiation compared to non-survivors (median 1382 versus 3265 ml; p = 0.003). Hourly fluid balance per bodyweight (FB^net^) was lower in survivors at 0–24, 24–48 and 48–72 h after initiation of CRRT (p < 0.008 for all comparisons). In the survival analysis (analyzed with counting process model) significant time-dependent explanatory variables for hospital mortality were FB^net^ (per ml/kg/h: HR: 1.319, 95% CI 1.038–1.677, p = 0.02), lactate (HR: 1.086, 95% CI 1.030–1.145, p = 0.002) and SOFA score (per ml/kg/h: HR: 1.084, 95% CI 1.025–1.146, p = 0.005) during the first 72 h of CRRT. Even after careful adjustment for repeated measures of disease severity, FB^net^ during the first 72 h of CRRT remains independently associated with hospital mortality, in critically ill patients with AKI.

## Introduction

Critically ill patients often require fluid resuscitation in the early phase of treatment, which can lead to fluid overload and a later need for fluid removal. Several observational studies have shown that fluid overload with coincident acute kidney injury (AKI) is associated with worsening organ dysfunction and increased mortality in critically ill patients^1–3^ and extracorporeal removal of excess fluid using renal replacement therapy (RRT) techniques is associated with reduced mortality^1, 4^.

However, previous data on the association between fluid balance and mortality may be confounded as the ability to remove fluid is highly dependent on the severity of acute illness and the capacity to maintain fluid homeostasis in critically ill AKI patients. Therefore, severity of illness and need for organ support may be more important determinants of mortality, whereas, fluid balance may serve as a mere surrogate marker. In line with this assumption, a recent survey of net ultrafiltration (nUF) prescription in Europe showed that in the occurrence of hemodynamic instability, defined as onset or worsening of tachycardia, hypotension or need to start or increase the dose of vasopressors, 70% of practitioners decreased the rate of fluid removal or even administered fluid boluses^5^. Moreover, in a previous large retrospective study by Shawwa et al. hypotension within one hour of CRRT initiation was associated with increased in-hospital mortality^6^. Notably, patients observed with incident early hypotension during CRRT had higher disease severity in terms of Sequential Organ Failure Assessment (SOFA) score, blood lactate and vasopressor dose compared to others.

The secondary analysis of 1434 patients of the “Randomized Evaluation of Normal versus Augmented Level of Renal Replacement Therapy” (RENAL) trial indicated that nUF rates of > 1.75 ml/kg/h increased mortality compared to nUF of < 1.01 ml/kg/h^7^ and this finding was later corroborated in another cohort study^8^. A further examination of the RENAL study data showed that the deleterious effects a high nUF rates compared to more modest rates were especially evident in the most severely ill patients.

Thus, whether fluid balance during early CRRT is a true determinant of mortality independent of disease severity in critically ill patients with AKI remains controversial. Previous studies have not included multivariable models with repeated measures of disease severity markers such as SOFA score, lactate level or vasopressor requirement during the first days following CRRT initiation as the models have only been adjusted with disease severity at the time of CRRT initiation.

Therefore, we aimed to study whether fluid balance during the first 72 h of CRRT is independently associated with hospital mortality after meticulous adjustment for repeated measures of disease severity during the time frame in a cohort of critically ill patients with AKI.

## Results

### Patient characteristics

A total of 493 patients required CRRT during the ICU care between January 2010 and December 2019. 86 patients with CRRT termination within 24 h and 8 patients on maintenance dialysis were excluded leaving 399 patients (115 women, 28.8%) with a mean age of 64.8 ± 13.1 years for the analyses (Supplemental Fig. S1). The most common comorbidities were hypertension (65.2%), diabetes (37.3%) and coronary artery disease (28.6%) (Table 1). Of the surgical patients 41.8% had undergone cardiac surgery, 24.8% gastroinstestinal surgery, 20.3% vascular surgery, 3.9% trauma surgery and 9.2% other miscellaneous surgery. Only 24.2% of the surgical operations were elective, whereas 75.8% were urgent or emergency surgery. The median FB^cum^ from ICU admission to initiation of CRRT was 1767 (193–5114) ml. FB^cum^ at CRRT initiation was associated with the time to CRRT initiation from ICU admission (r = 0.67, p < 0.0001) (Supplemental Fig. S2). Almost all patients (93.0%) required vasopressor support at ICU admission and 77.9% at CRRT initiation and 72.7% required mechanical ventilation during ICU stay. Median duration of mechanical ventilation was 7.7 (3.8–12.8) days and tracheostomy was required in 68 (17.1%) patients. Hospital mortality was 33.6% (134 patients). (Table 1) Altogether, 26%, 45% and 60% of the patients had negative FB^net^ during the first 24 h, 24–48 h and 48–72 h of CRRT, respectively. Hospital survivors had higher hourly diuresis at 24 and 72 h from CRRT initiation compared with non-survivors (Supplemental Fig. S3). Comparisons between patients achieving a negative fluid balance at least on one day during first 72 h of CRRT and patients not achieving negative daily fluid balance are shown in Supplemental Table S1.

**Table 1:** Baseline patient characteristics and mortality of the 399 patients.

Women (n/%)	115/28.8
Age (years)	64.8 ± 13.1
BMI (kg/m^2^)	27.8 (24.6–33.1)
Non-surgical patients (n/%)	246/61.7
Hypertension (n/%)	260/65
Diabetes (n/%)	149/37
Atrial fibrillation (n/%)	95/24
History of stroke (n/%)	37/9
Heart failure (n/%)	98/25
Pulmorary disease (n/%)	57/14
Coronary artery disease (n/%)	114/29
Peripheral arterial disease (n/%)	49/12
Liver cirrhosis (n/%)	7/2
Malignancy (n/%)	29/7
Baseline creatinine, n = 335 (µmol/l)	87 (66–128)
Baseline eGFR, n = 335 (ml/min/1.73m^2^)	81 (62–97)
SOFA on admission	10 (7–12)
Peak SOFA	14 (11–16)
SAPS-II on admission	54 (45–64)
APACHE-II on admission	25 (21–30)
Time to CRRT from ICU admission (h)	13 (3–36)
Noradrenaline requirement at start of dialysis (µg/kg/min)	0.11 (0.03–0.20)
Mean arterial pressure at start of dialysis (mmHg)	71 (64–82)
Hourly urine output at start of dialysis (ml)	11.5 (4.4–31.5)
Dialysis dose (ml/kg/h)	34.2 (30.0–36.4)
ICU fluid balance at start of dialysis (ml)	1767 (193–5114)
Duration of CRRT (h)	107 (67–235)
ICU stay (days, survivors, n = 298)	8.4 (4.4–15.6)
Vasopressor use (n/%)	371/93.0
Vasopressor requirement 24 h after CRRT initiation (n/%)	277/69
Vasopressor requirement 48 h after CRRT initiation (n/%)	201/59
Vasopressor requirement 72 h after CRRT initiation (n/%)	145/48
Mechanical ventilation (n/%)	290/72.7
Days on mechanical ventilation (days)	7.7 (3.8–12.8)
Tracheostomy during ICU stay (n/%)	68/17.1
ICU mortality (n/%)	101/25.3
Hospital mortality (n/%)	134/33.6

Values are mean ± SD or median (IQR).

*BMI* body mass index, *eGFR* Estimated Glomerular Filtration Rate, *SOFA* Sequential Organ Failure Assessment Score, *SAPS-II* Simplified Acute Physiology Score, *APACHE-II* Acute Physiology and Chronic Health Evaluation Score II, *CRRT* Continuous Renal Replacement Therapy, *ICU* Intensive Care Unit.

The most frequent causes of AKI were septic AKI (31.8%), post cardiac surgery AKI (16.0%), prerenal/renal non-septic AKI (15.3%), post gastrointestinal surgery AKI (11.0%), post vascular surgery AKI (8.0%) and cardiorenal AKI (5.3%). The most frequent causes of hospital death were infection (37.3%), cardiovascular (40.3%), gastrointestinal (11.9%) and traumatological (3.0%). Hospital survivors were more often non-surgical but had a similar incidence of mechanical ventilation during ICU stay compared to patients who deceased during hospital care. There were no differences in pH, CRP, hemoglobin, leukocytes, platelets, urea, sodium, potassium, base excess, bicarbonate, chloride, ionized calcium or mean arterial pressure at the time of CRRT initiation between survivors and non-survivors. However, non-survivors had lower creatinine, platelets and BMI and higher age, time to CRRT initiation, SOFA, SAPS-II, APACHE-II, blood lactate, bilirubin, international normalized ratio, norepinephrine dose, number of required vasopressors and maximum norepinephrine dose at time of CRRT initiation (Table 2).

**Table 2:** Characteristics of hospital survivors compared to non-survivors.

Variable	Survivors (n = 265)	Nonsurvivors (n = 134)	p-value
Age (years)	63.2 ± 13.2	68.0 ± 12.2	** < 0.001**
Women n/%	76/19	39/10	0.93
BMI (kg/m^2^)	29.9 ± 7.0	28.0 ± 6.0	**0.007**
Time to CRRT initiation (h)	10 (3–33)	18 (5–47)	**0.001**
SOFA	11 (9–13)	13 (11–14)	** < 0.001**
SAPS-II	52 (43–62)	60 (50–70)	** < 0.001**
APACHE-II	25 (20–30)	26 (22–32)	**0.007**
Non-surgical patients (n/%)	175/66.0	71/53.0	**0.01**
Mechanical ventilation requirement (n/%)	173 (65.3)	117 (87.3)	0.25
FB^cum^ at start of dialysis (ml)	1382 (135–4152)	3265 (432–6150)	**0.003**
FB^cum^ 0–24 h after start of dialysis (ml)	1229 (− 354–2963)	1855 (376–3878)	**0.004**
FB^cum^ 24–48 h after start of dialysis (ml)	1270 (− 841–4406)	2009 (− 156–5933)	**0.03**
FB^cum^ 48–72 h after start of dialysis (ml)	1983 (− 1812–7600)	3659 (87–10,346)	**0.04**
FB^net^ 0–24 h (ml/kg/h)	0.54 (− 0.15–1.50)	0.94 (0.19–2.07)	**0.001**
FB^net^ 24–48 h (ml/kg/h)	0.02 (− 0.36–0.67)	0.22 (− 0.21–1.15)	**0.007**
FB^net^ 48–72 h (ml/kg/h)	− 0.23 (− 0.56–0.13)	0.03 (− 0.44–0.54)	**0.003**
Fluid overload > 5% of TBW (n/%)	61/23	56/42	** < 0.001**
Fluid overload > 10% of TBW (n/%)	26/10	24/18	**0.02**
Norepinephrine requirement 24 h (n/%)	165/62	111/83	** < 0.001**
Norepinephrine requirement 48 h (n/%)	126/53	75/73	** < 0.001**
Norepinephrine requirement 72 h (n/%)	84/39	61/67	** < 0.001**
pH	7.30 (7.21–7.37)	7.29 (7.23–7.36)	0.80
Creatinine (µmol/l)	336 (230–495)	274 (197–380)	**0.001**
Urea (mmol/l)	20.2 (12.8–28.2)	19.7 (13.5–30.6)	0.97
CRP (mg/l)	120 (39–235)	133 (51–226)	0.74
Hemoglobin (g/l)	103 (91–120)	101 (91–114)	0.27
Leukocytes (10^9^/l)	14 (9–20)	13 (9–20)	0.67
Platelets (10^9^/l)	158 (92–242)	98 (65–173)	** < 0.001**
International normalized ratio	1.3 (1.1–1.7)	1.8 (1.3–2.3)	** < 0.001**
Bilirubin (µmol/l)	12 (7–24)	20 (10–43)	** < 0.001**
Lactate (mmol/l)	1.63 (1.01–3.79)	2.87 (1.42–6.03)	** < 0.001**
Sodium (mmol/l)	137 (134–139)	136 (134–139)	0.62
Potassium (mmol/l)	4.1 (3.7–4.6)	4.2 (3.9–4.6)	0.09
Mean arterial pressure (mmHg)	71 (63–83)	68 (62–79)	0.24
Norepinephrine dose (µg/kg/min)	0.10 (0.01–0.19)	0.15 (0.05–0.25)	**0.001**
Number of vasopressors (n)	1 (1–1)	1 (1–2)	**0.03**
Maximum norepinephrine dose (µg/kg/min)	0.20 (0.13–0.33)	0.33 (0.20–0.53)	** < 0.001**
BE	− 6.6 (− 11.6 to − 3.7)	− 7.6 (− 10.9 to − 4.2)	0.49
Bicarbonate (mmol/l)	19.1 (15.5–21.2)	18.2 (15.8–21.0)	0.47
Chloride (mmol/l)	107 (103–111)	108 (105–108)	0.13
Ionized calcium (mmol/l)	0.98 (0.91–1.03)	0.96 (0.89–1.02)	0.17

Values are mean ± SD or median (IQR).

Values are given at CRRT initiation, unless stated otherwise.

*BMI* Body Mass Index, *CRRT* Continuous Renal Replacement Therapy, *SOFA* Sequential Organ Failure Assessment Score, *SAPS-II* Simplified Acute Physiology Score, *APACHE-II* Acute Physiology And Chronic Health Evaluation Score II, *FB*^*cum*^ Cumulative Fluid Balance, *FB*^*net*^ Mean Hourly Fluid Balance Per Bodyweight; *CRP* C-reactive Protein, *BE* Base Excess.

**Figure 1:**
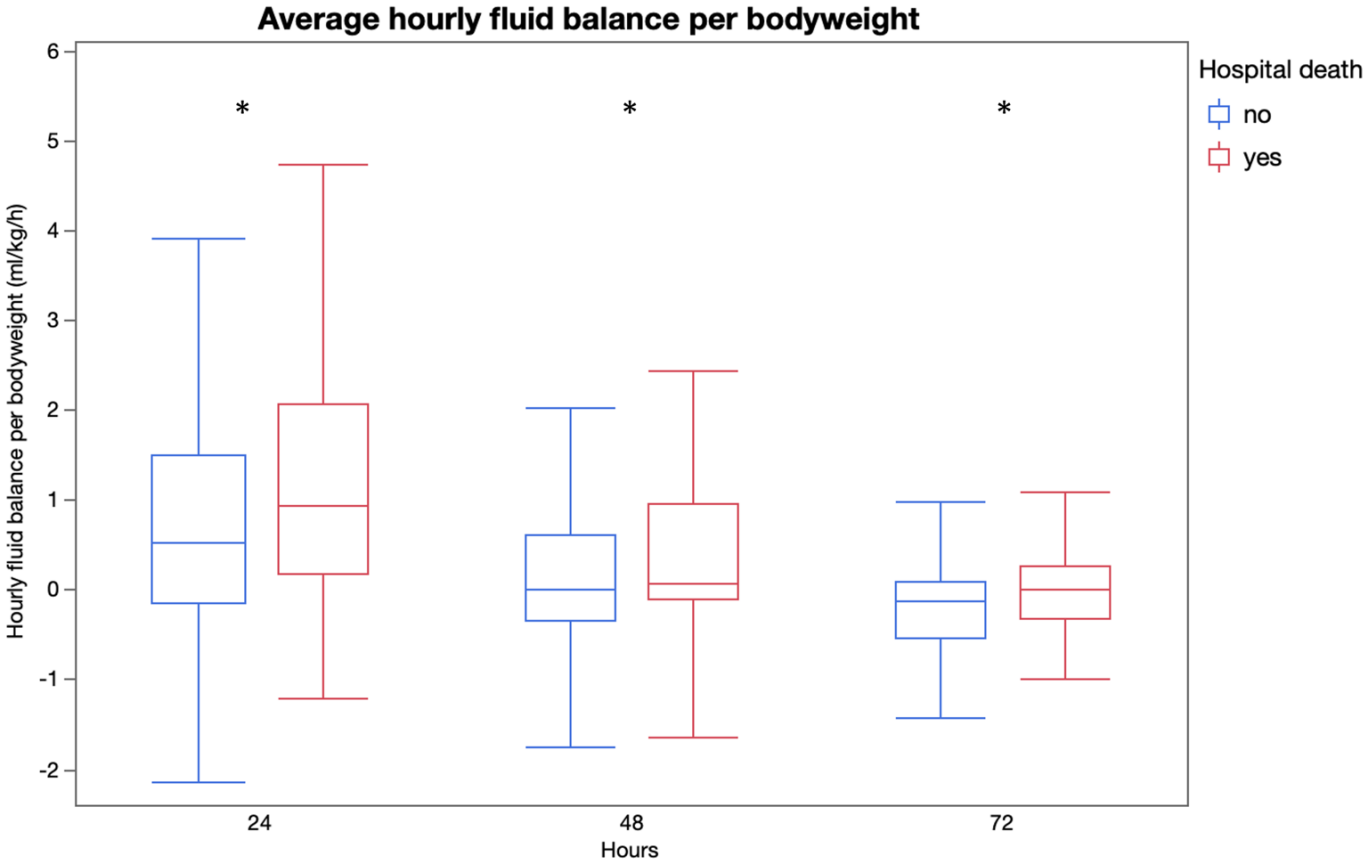
Hourly fluid balance by time (*p < 0.01, **p < 0.001). The figure was generated using JMP Pro 15.1.0 software. Copyright 2019 © SAS Institute Inc. JMP Pro and all other SAS Institute Inc. product or service names are registered trademarks or trademarks of SAS Institute Inc., Cary, NC, USA.

**Figure 2:**
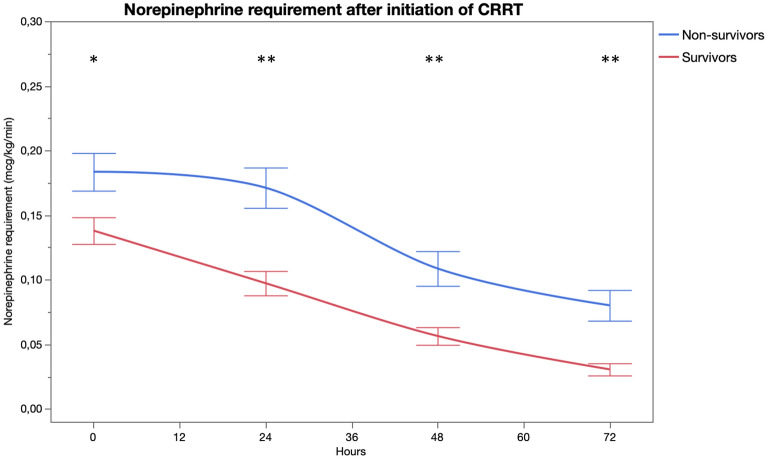
Norepinephrine requirement by time (*p < 0.01, **p < 0.001). The figure was generated using JMP Pro 15.1.0 software. Copyright 2019 © SAS Institute Inc. JMP Pro and all other SAS Institute Inc. product or service names are registered trademarks or trademarks of SAS Institute Inc., Cary, NC, USA.

**Figure 3:**
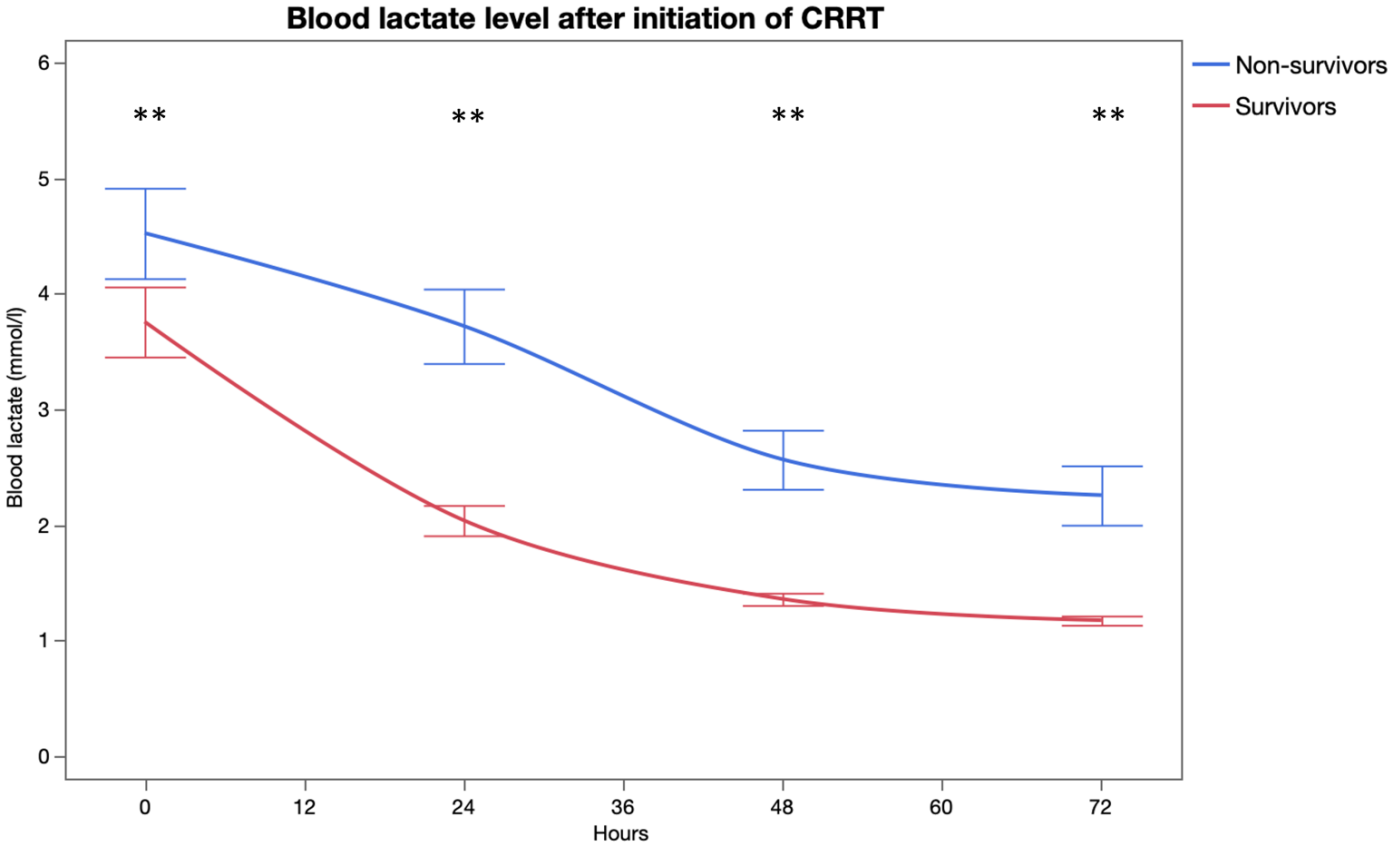
Lactate by time (*p < 0.01, **p < 0.001). The figure was generated using JMP Pro 15.1.0 software. Copyright 2019 © SAS Institute Inc. JMP Pro and all other SAS Institute Inc. product or service names are registered trademarks or trademarks of SAS Institute Inc., Cary, NC, USA.

**Figure 4:**
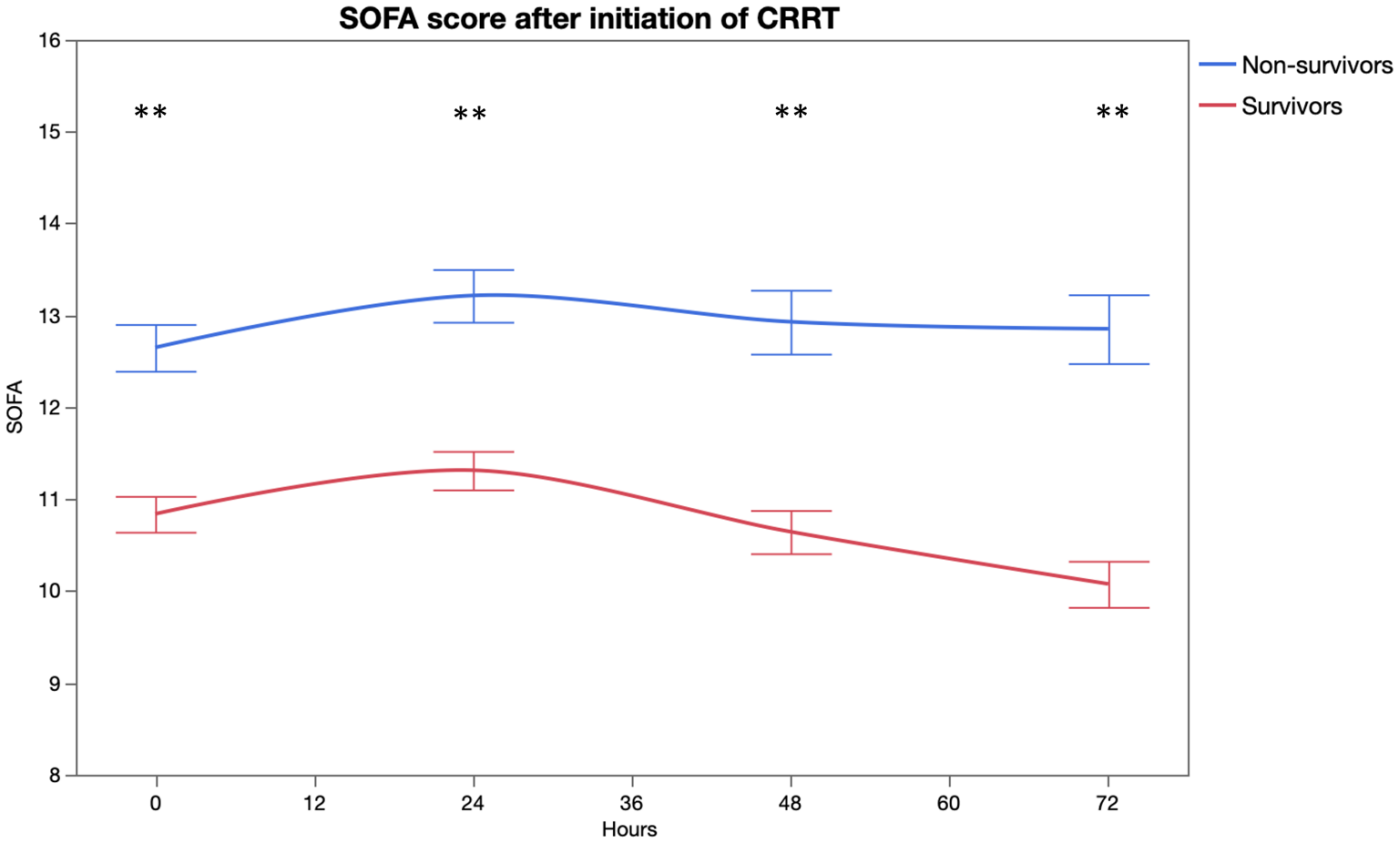
SOFA score by time (*p < 0.01, **p < 0.001). The figure was generated using JMP Pro 15.1.0 software. Copyright 2019 © SAS Institute Inc. JMP Pro and all other SAS Institute Inc. product or service names are registered trademarks or trademarks of SAS Institute Inc., Cary, NC, USA.

Survivors had lower FB^cum^ at CRRT initiation and a lower incidence of significant fluid overload compared to non-survivors. Furthermore, FB^cum^ (Table 2), FB^net^ (Fig. 1), norepinephrine requirement (Fig. 2), lactate (Fig. 3) and SOFA scores (Fig. 4) differed significantly between the groups at 24, 48 and 72 h after initiation of dialysis.

### Fluid balance, severity of illness and mortality

The repeated time-dependent FB^cum^ at CRRT initiation and at 24 h, 48 h and 72 h after initiation was associated with mortality in the univariate counting process model (per litre: β = 0.031, HR: 1.031, 95% CI 1.008–1.054, p = 0.007). Moreover, all the severity of illness markers: lactate (β = 0.153, HR: 1.165, 95% CI 1.126–1.205, p < 0.0001), SOFA score (β = 0.122, HR: 1.130, 95% CI 1.078–1.184, p < 0.0001) and noradrenalin dose (per 0.1 µmol/kg/min: β = 0.219, HR: 1.245, 95% CI 1.134–1.367, p < 0.0001) and age (per 1 year: β = 0.0021, HR: 1.021, 95% CI 1.006–1.036, p = 0.005) were associated with mortality in the univariate models. In the multivariable counting process model the association between mortality and FB^cum^ became nonsignificant (β = 0.0002, HR: 1.002, 95% CI 0.986–1.017, p = 0.85) and FB^cum^ was eliminated from the final model, whereas, the association remained significant for lactate (β = 0.18, HR: 1.194, 95% CI 1.132–1.259, p < 0.0001), SOFA score (β = 0.12, HR: 1.129, 95% CI 1.069–1.194, p < 0.0001) and noradrenalin dose (per 0.1 µmol/kg/min: β = 0.15, HR: 1.163, 95% CI 1.026–1.318, p = 0.02).

The repeated time-dependent FB^net^ during the first 24 h, 24–48 h and 48–72 h after CRRT initiation was associated with mortality in the univariate counting process model (per ml/kg/h: β = 0.42, HR: 1.529, 95% CI 1.242–1.881, p < 0.0001). In the multivariable model significant time-dependent explanatory variables for hospital mortality were FB^net^ (per ml/kg/h: β = 0.28, HR: 1.353, 95% CI 1.038–1.677, p = 0.02), lactate (β = 0.08, HR: 1.086, 95% CI 1.030–1.145, p = 0.002) and SOFA score (β = 0.08, HR: 1.084, 95% CI 1.025–1.146, p = 0.005). The association between FB^net^ and hospital mortality remained significant (per ml/kg/h: β = 0.30, HR: 1.353, 95% CI 1.063–1.722, p = 0.01) when FB^cum^ at CRRT initiation was included as a covariate in the multivariable model. However, FB^cum^ at CRRT initiation was not significantly associated with mortality in the multivariable model (per litre: β = 0.03, HR: 1.032, 95% CI 0.989–1.077, p = 0.15). The univariate and multivariate associations between repeated measures of disease severity indices and FB^net^ and FB^cum^ at CRRT initiation and hospital mortality are shown in Supplemental Table S2.

When the association between hospital mortality and FB^net^ was examined using respective Cox models at the first 24 h, 48 h and 72 h of CRRT adjusted for the corresponding lactate, SOFA score and noradrenaline dose and age with backward elimination, the association between FB^net^ and mortality remained significant at each time point (24 h: β = 0.21, HR: 1.238, 95% CI 1.104–1.387, p = 0.0003; 48 h: β = 0.26, HR: 1.293, 95% CI 1.063–1.572, p = 0.001; 72 h: β = 0.34, HR: 1.398, 95% CI 1.072–1.823, p = 0.01).

Ultrafiltration was also significantly associated with hospital mortality in a univariate counting process model (per ml/h: β =  − 0.005, HR: 0.995, 95% CI0.991–0.999, p = 0.02), but not after adjustment for SOFA score, lactate, noradrenalin dose and age in a multivariable model (p = 0.55).

## Discussion

Our study shows that higher FB^net^ during the first 72 h of CRRT is independently associated with hospital mortality in critically ill patients with CRRT dependent AKI, after adjustment for repeated measures of coincident markers of disease severity during the time frame. Compared to previous studies in the field, our current data and findings emphasize the independent association between FB^net^ during early days of CRRT and mortality, which has so far remained controversial.

Prior data on the association between fluid balance and mortality may have been partly confounded as the ability to remove fluid is highly dependent on the severity of acute illness and the patient’s capacity for fluid homeostasis control. To our knowledge this is the first study evaluating the effect of several repeated measures of FB^net^ and coincident severity of illness markers on hospital mortality in critically ill AKI patients receiving CRRT.

A prospective study by Bouchard et al. (2009) showed that fluid overload exceeding 10% of bodyweight on the day of RRT initiation was associated with an increased risk of mortality after adjustment for APACHE-III score at the time of the AKI diagnosis and RRT modality^1^. In a Finnish prospective follow-up study cumulative fluid overload at RRT initiation was associated with increased 90-day mortality, and the association remained significant after adjustment for disease severity at the time of RRT initiation, time to RRT initiation, initial RRT modality, patient's sepsis status, and several other parameters^2^. In the study by Vaara et al. (2012) the majority of non-survivors with fluid overload died in the hospital within a short time period, implying a severe course of disease. In a more recent retrospective study of 341 critically ill patients with AKI requiring CRRT, sepsis and/or high SOFA score and total fluid overload > 10% of body weight, observed from 3 days before CRRT initiation to ICU discharge, had lower ICU survival compared to patients without fluid overload^9^. However, this finding was limited to patients with sepsis and high SOFA scores and not observed in patients with less severe illness. In the present study, FB^cum^ measured at CRRT initiation and repeatedly during the first 72 h of CRRT was associated with hospital mortality in the univariate model, but the association did not remain significant after controlling for repeated measures of disease severity markers and age. This finding emphasizes the importance of using repeated measures of severity of illness when assessing the independent effects of fluid balance on patient prognosis.

Most recent studies on fluid balance in patients with RRT dependent AKI have focused on the association between fluid removal rate (namely nUF rate) during RRT and mortality^5, 7, 8, 10–12^. The RENAL trial data indicated that nUF rates of > 1.75 ml/kg/h increased mortality compared to a nUF rate of < 1.01 ml/kg/h^7^ and this finding was later reproduced in another cohort study^8^. A further examination of the RENAL study data showed that the deleterious effects a high nUF rates compared to mediocre rates (1.01–1.75 ml/kg/h) are especially evident in patients with the highest severity of illness. It is notable that in the RENAL study patients considered to have a mediocre nUF rate (1.01–1.75 ml/kg/h) had a median daily fluid balance of only − 55 ml/day from RRT initiation to ICU discharge and patients with high nUF rate (> 1.75 ml/kg/h) had a median daily fluid balance of − 658 ml/day corresponding to FB^net^ of − 0.03 ml/kg/h and − 0.34 ml/kg/h, respectively^7^. Therefore, it would advisable to assess FB^net^ in AKI patients on RRT, instead of nUF rates as only FB^net^ gives a realistic understanding of the development of weight adjusted fluid status during RRT.

Only a few recent studies have assessed the development of fluid balance during the first days of CRRT and its association with mortality. Jhee and colleagues (2019) studied retrospectively the impact of FB^cum^ on short term mortality in 258 critically ill patients requiring CRRT^13^ and found that an increase in FB^cum^ during the first 24 and 72 h from CRRT initiation were associated with increased 7 and 28 day mortality. The association between FB^cum^ and mortality was examined using respective Cox proportional hazards models for both time points (24 h and 72 h) and the analyses were adjusted for MAP, hemoglobin, SOFA score, use of vasopressor, and type of fluid administration at both time points, respectively. Dynamic analysis of the within subject time-dependent development of FB^cum^, lactate, vasopressor dose and SOFA scores was not employed and the study had only 2/3 of the sample size of the present study. Naorungroj et al. (2020) retrospectively evaluated hourly fluid balance for the first 48 h of CRRT in 350 patients^14^. Hourly monitoring of fluid balance allowed using the area under the curve for the evaluation of the development of fluid balance and demonstrated that the percentage of negative hourly fluid balance during the first 48 h of CRRT was independently associated with decreased ICU mortality. However, negative fluid balance was never achieved in patients receiving vasoactive therapy in the reference study. In our study 78%, 69%, 59% and 48% of patients required vasopressors at CRRT initiation and at 24, 48 and 72 h of CRRT, respectively, indicating that the patients in our study cohort were severely ill, but negative FB^net^ was, nevertheless, achieved in 45% of patients by 48 h and 60% of patients by 72 h from CRRT initiation.

A recent large study of Hall et al. (2020) retrospectively evaluated the association of the development of FB^cum^ during CRRT on mortality of 820 critically ill AKI patients^15^. Not reaching any reduction in FB^cum^ during CRRT was associated with hospital mortality after adjustment for demographics, SOFA score and lactate on the day of CRRT initiation and mean daily noradrenaline dose during CRRT. When patients that did not reach a reduction in FB^cum^ during CRRT were excluded from the analyses, the delta FB^cum^ (defined as FB^cum^ at CRRT initiation – the lowest value of FB^cum^ during CRRT) was associated with hospital mortality, whereas, FB^cum^ at CRRT initiation was not. However, although FB^cum^ was assessed daily for the first 7 days of CRRT, markers of disease severity (SOFA score and lactate) were only assessed once on the day of CRRT initiation.

It is not clear, why FB^cum^ at the start and during the first 72 h of CRRT was not independently associated with mortality after adjustment with repeated measures of disease severity in the current study in contrast to FB^net^. It is plausible that this finding resulted from varying time intervals from ICU admission to CRRT initiation, with longer ICU stays prior to CRRT initiation probably leading to increased fluid overload in affected individuals. Furthermore, using a variable such as FB^cum^ with a high within subject correlation over the time frame of interest may have influenced the statistical power of the model. Above all, however, it seems that the incorporation of body weight in the equation for FB^net^ is probably of clinical relevance as is the development of fluid balance after commencing CRRT irrespective FB^cum^ at CRRT initiation. Moreover, the association between FB^net^ during CRRT and hospital mortality remained significant after including FB^cum^ at CRRT initiation in the multivariable model.

Our current findings have clinical implications. Our data show that strict control of fluid balance during the first days of CRRT are essential in spite of disease severity and need for vasopressors. In line with this conclusion, only a minor proportion of patients in the current study had significant fluid overload at CRRT initiation and a negative FB^net^ was still reached in 60% of the patients by the third day of CRRT. The primary focus of fluid balance management should be a daily fluid balance prescription which is implemented by the means of restrictive fluid administration and continuous electronic measurement of fluid balance to guide flexible adjustments of nUF to meet the daily fluid balance goal.

The limitations of this study pertain to its retrospective design. Since data of the current study were collected at a single center, the results may not apply to other institutions. Due to the restrictive fluid resuscitation and relatively early initiation of RRT policies employed at our center only a minor proportion of the study patients had significant > 10% fluid overload at CRRT initiation. Still FB^net^ was associated with mortality after adjusting for disease severity, which, only emphasizes the importance of fluid balance control during early CRRT. Patients that deceased within 24 h of CRRT initiation were excluded from the study, which decreases the mortality rate of our study population. Similarly to many other studies on this subject, we were not able to measure the fluid input that patients had received before ICU admission, which could be a valuable addition to future studies but requires firm data collection especially for patients admitted to the ICU from other healthcare units. However, the ICU patients at our center are documented very meticulously, and the problem of missing values is rather nonexistent for the ICU period. Furthermore, we included only patients that required CRRT for AKI and all patients were treated with citrate-calcium anticoagulation. Moreover, we used repeated daily measures of critical illness markers and fluid balance for the statistical analyses, which makes this study to our knowledge unique in its design.

## Conclusions

FB^net^ assessed as a repeated measure during the first 72 h of CRRT is associated with increased hospital mortality even after adjustment for repeated measures of disease severity markers in critically ill patients with AKI. Our results suggest that positive fluid balance during the early days of CRRT is detrimental.

## Methods

### Data sources, collection and study population

Patients admitted to a single intensive care unit (ICU) of an academic tertiary medical center from January 1, 2010 through December 31, 2019 and requiring CRRT at least for 24 h were included in this retrospective cohort study. Patients receiving CRRT less than 24 h and patients on maintenance dialysis before ICU admission were excluded from the analyses.

The individual patient data were collected from the hospital’s medical documents. For the purpose of this study, blood lactate, pH, bicarbonate, base excess, electrolytes, and other laboratory variables, need for invasive mechanical ventilation, PaO2/FiO2-ratio, diuresis, vasopressors, Acute Physiology and Chronic Health Evaluation (APACHE-II) II score, Simplified Acute Physiology Score (SAPS-II) II, and SOFA score were recorded at ICU admission, at CRRT initiation and 24, 48 and 72 h after CRRT initiation. Cumulative fluid balance (FB^cum^) from ICU admission together with 5% and 10% fluid overload (fluid accumulation in reference to the baseline weight recorded at ICU admission) were recorded from ICU admission to CRRT initiation. Furthermore, FB^cum^ and the mean hourly fluid balance per bodyweight (FB^net^) were recorded at 24, 48 and 72 h after CRRT initiation. Balance calculations were performed based on total fluid intake from all sources (intravenous or ingested fluids, blood products, medications and enteral and/or parenteral nutrition) minus all outputs (urine, CRRT nUF, drain losses and gastrointestinal output). Hourly urine output, ultrafiltration, mean arterial pressure (MAP) and vasoactive therapy requirement rate were recorded at CRRT initiation and 24, 48 and 72 h after CRRT initiation. For the assessment of baseline renal function, creatinine and eGFR within one year prior to ICU admission were recorded when available. Other data extracted from patients’ medical records included demographics and chronic medical conditions.

### CRRT modality

Continuous Veno-Venous Hemodialysis for all patients was performed using Fresenius Multifiltrate CRRT monitors and 1.80 m^2^ polysulfone hemofilters Ultraflux AV1000 or Ultraflux EMiC2 HCO membranes with CiCa dialysate to achieve regional citrate anticoagulation (Fresenius Medical Care, Bad Hamburg, Germany). Post-filter-ionized calcium levels were used for anticoagulation monitoring. Blood and dialysate flow rates were prescribed according to the weight of the patient and by the treating ICU physician to target a dialysis dose of > 25 ml/kg/h. The methodology for CRRT remained unaltered for the entire study period. At our center fluid balance is prescribed daily by the attending physician and in CRRT patients, nUF rate is titrated continuously by the registered CRRT nurses to meet the daily balance prescription.

### Statistical analysis

Results are presented as mean and standard deviation (SD) for the normally distributed variables and as median inter-quartile range (IQR) for skewed variables. For the skewed variables, we examined different transformations (log e-transformation, square root transformation and square transformation) to normalize distributions, and the best transformation for each variable was chosen according to tests for normality (Shapiro–Wilk, Kolmogorov–Smirnov) and visual examination. Student’s t-test was used to compare continuous normally distributed covariates and Chi-square test for categorical covariates in the study subgroups (hospital survivors vs. non-survivors). For variables with skewed distributions and without acceptable transformations, groupwise comparisons were done using a non-parametric Kruskall-Wallis test.

Univariate and multivariable associations between repeated measures of the markers of acute disease severity (SOFA score, vasopressor dose and lactate), FB^cum^ or FB^net^, respectively, at CRRT initiation and during the first 72 h of CRRT (time-dependent explanatory variables), age and hospital mortality were assessed with survival analysis method using a counting process method with time intervals of 24 h. Furthermore, the association between FB^net^ during CRRT and hospital mortality with adjusted for markers of disease severity and age was examined using respective Cox proportional hazards models at time points 24 h, 48 h and 72 h after CRRT initiation. For the multivariable analyses, all the aforementioned covariates were first included in the multivariable models followed by removing the least significant variables one at a time to yield the final multivariable models including only significant adjusted variables. Hazard ratios together with 95% confidence intervals from the models are reported.

All statistical analyses were performed using statistical analysis system, SAS version 9.4 (SAS Institute Inc., Cary NC). P < 0.05 (two-tailed) was considered statistically significant.

### Ethics declarations

The study protocol was approved by the Turku University Clinical Research Center scientific review board (Turku CRC) and the Hospital district of Southwest Finland (Reference number: T143/2019). The patient identity numbers were removed and the hospital software data combined before the statistical analyses. For this retrospective, register-based, non-interventional study the Turku CRC and Hospital district of Southwest Finland joint review board waived the need for informed consent in terms of data collection and analysis and publication of results. All experiments were performed in accordance with relevant (STROBE) guidelines and regulations and all methods were carried out in accordance with relevant guidelines and regulations.

### Consent for publication

All data was anonymized, and this study does not contain any individual person’s data in any form (including individual details, images or videos). Therefore, consent for publication was waived.

## Supplementary Information

Supplementary Figure S1.

Supplementary Figure S2.

Supplementary Figure S3.

Supplementary Tables.

## Data Availability

The data underlying this study contain potentially identifying participant information and cannot be shared publicly. Future data access requests should be sent to the Ethics Committee of Southwest Finland Hospital District (eettinen.toimikunta@tyks.fi) or the Department of Anesthesiology and Intensive Care and the Informatics Department of Turku University Hospital via the corresponding author.
