# Supplementary material for: Early restrictive fluid balance is associated with lower hospital mortality independent of acute disease severity in critically ill patients on CRRT
Source: Sci Rep. 2021 Sep 14;11:18216. doi: 10.1038/s41598-021-97888-y (PMC8440636; doi:10.1038/s41598-021-97888-y)

# Supplemental Figure S1. Flow diagram of the study

The figure was generated using JMP Pro 15.1.0 software. Copyright 2019 © SAS Institute Inc. JMP Pro and all other SAS Institute Inc. product or service names are registered trademarks or trademarks of SAS Institute Inc., Cary, NC, USA.

## Flow Diagram

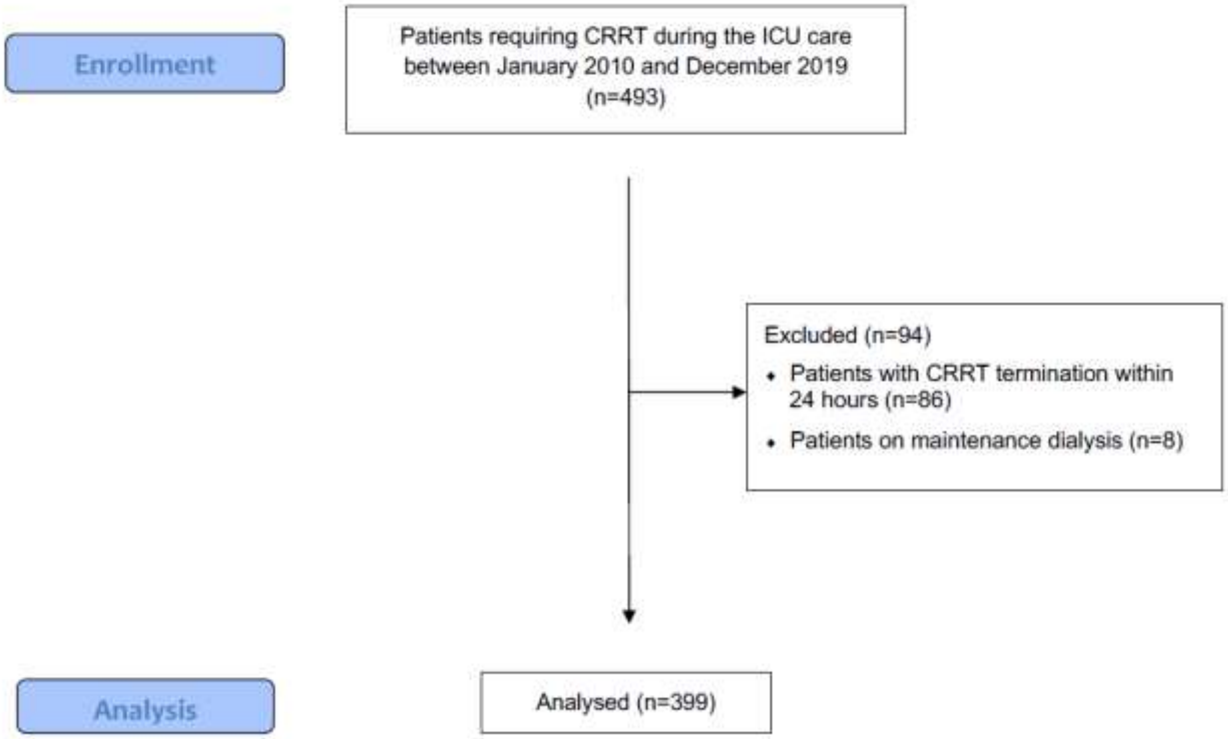

Supplement: Supplementary file 1 — Supplementary Figure S1. [file 41598_2021_97888_MOESM1_ESM.pdf]
