# Supplementary material for: Early restrictive fluid balance is associated with lower hospital mortality independent of acute disease severity in critically ill patients on CRRT
Source: Sci Rep. 2021 Sep 14;11:18216. doi: 10.1038/s41598-021-97888-y (PMC8440636; doi:10.1038/s41598-021-97888-y)
Supplement: Supplementary file 2 — Supplementary Figure S2. [file 41598_2021_97888_MOESM2_ESM.pdf]

Supplemental Figure S2. Association between cumulative fluid balance and time to CRRT initiation from ICU admission.

The figure was generated using JMP Pro 15.1.0 software. Copyright 2019 © SAS Institute Inc. JMP Pro and all other SAS Institute Inc. product or service names are registered trademarks or trademarks of SAS Institute Inc., Cary, NC, USA.

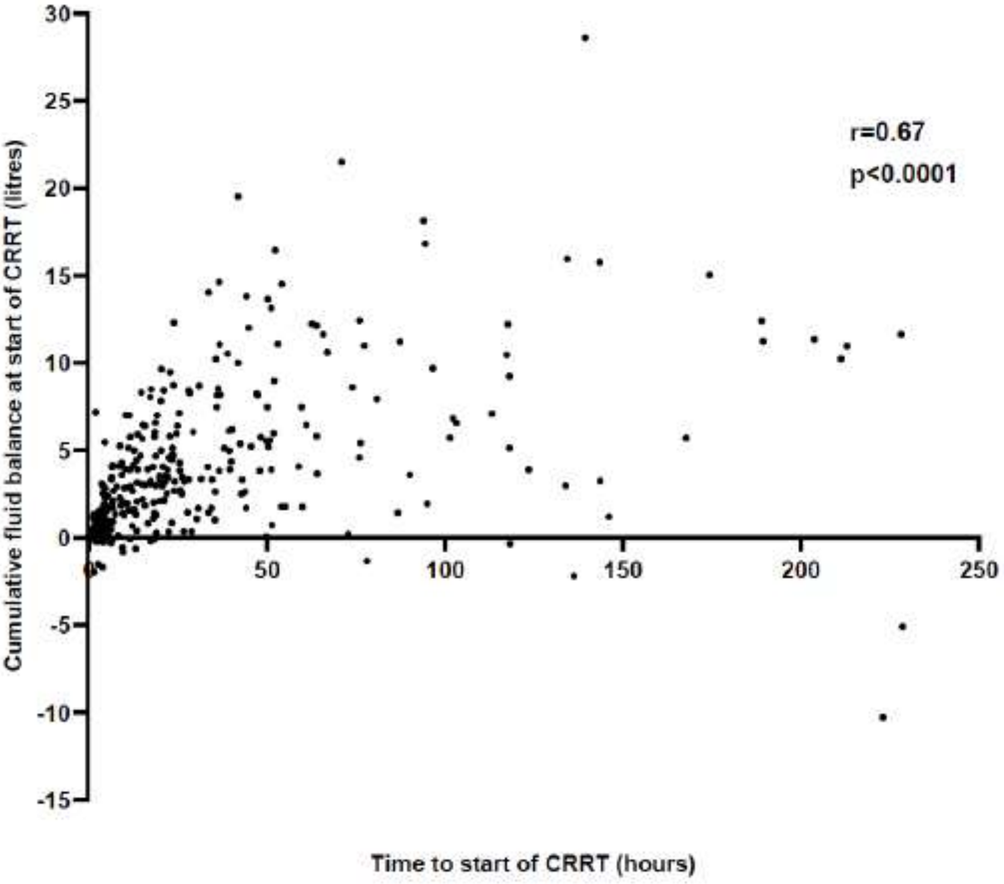

30
25
20
15
10
5
0
-5
-10
-15
0
50
100
150
200
250
Cumulative fluid balance at start of CRRT (litres)
r=0.67
p<0.0001
Time to start of CRRT (hours)
