# Supplementary material for: Early restrictive fluid balance is associated with lower hospital mortality independent of acute disease severity in critically ill patients on CRRT
Source: Sci Rep. 2021 Sep 14;11:18216. doi: 10.1038/s41598-021-97888-y (PMC8440636; doi:10.1038/s41598-021-97888-y)
Supplement: Supplementary file 3 — Supplementary Figure S3. [file 41598_2021_97888_MOESM3_ESM.pdf]

Supplemental Figure 3. Average hourly diuresis after initiation of CRRT.

The figure was generated using JMP Pro 15.1.0 software. Copyright 2019 © SAS Institute Inc. JMP Pro and all other SAS Institute Inc. product or service names are registered trademarks or trademarks of SAS Institute Inc., Cary, NC, USA.

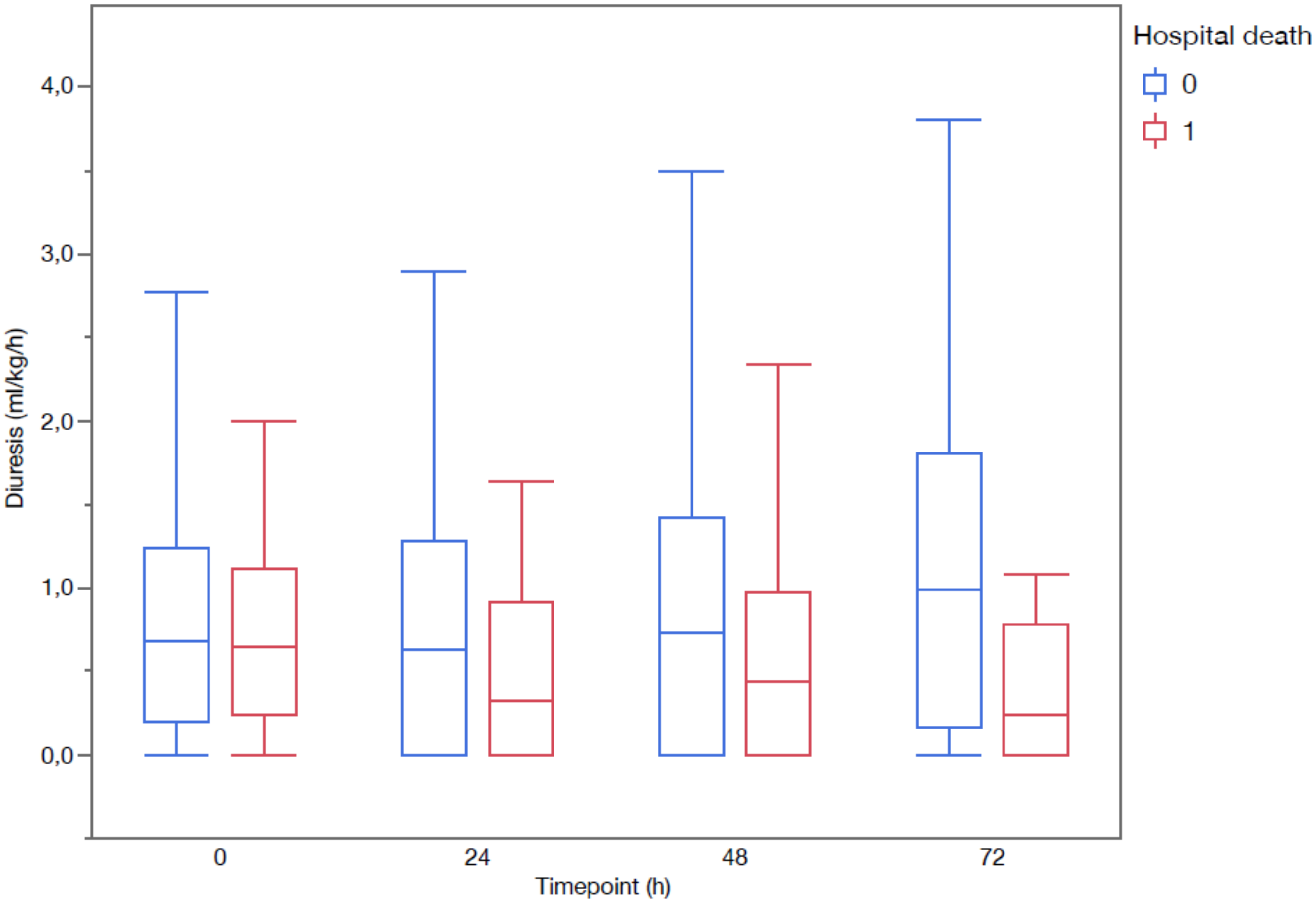
Hospital death
0
1
4,0
3,0
2,0
1,0
0,0
Diuresis (ml/kg/h)
0
24
48
72
Timepoint (h)
