# Supplementary material for: Early restrictive fluid balance is associated with lower hospital mortality independent of acute disease severity in critically ill patients on CRRT
Source: Sci Rep. 2021 Sep 14;11:18216. doi: 10.1038/s41598-021-97888-y (PMC8440636; doi:10.1038/s41598-021-97888-y)
Supplement: Supplementary file 4 — Supplementary Tables. [file 41598_2021_97888_MOESM4_ESM.docx]

**Supplemental Tables S1 and S2.**

| **Supplemental Table S1.** Characteristics of patients achieving a negative daily fluid balance during the first 72 hours of CRRT compared to those not achieving negative balance. Values are mean±SD or median (IQR). | | | | |  |  |
| --- | --- | --- | --- | --- | --- | --- |
| **Variable** | **Negative daily balance (n=250)** | | **Positive daily balance (n=149)** | **p-value** | |  |
| Age (years) | | | 69 (59-74) | 66 (57-72) | 0.21 | |
| Women n/% | | | 70/28 | 45/30 | 0.64 | |
| BMI (kg/m^2^) | | | 28.1 (24.8-33.7) | 27.6 (24.5-31.2) | 0.11 | |
| Time to CRRT initiation (h) | | | 18 (3-47) | 6 (3-21) | **<0.001** | |
| SOFA | | | 11 (9-13) | 12 (10-14) | **0.002** | |
| SAPS-II | | | 52 (44-62) | 60 (48-70) | **<0.001** | |
| APACHE-II | | | 25 (21-29) | 26 (21-31) | **0.04** | |
| Non-surgical patients (n/%) | | | 150/60 | 96/64 | 0.38 | |
| Mechanical ventilation requirement (n/%) | | | 174 (70) | 116 (78) | 0.07 | |
| FB^cum^ at start of dialysis (ml) | | | 2023 (158-5527) | 1550 (377-4139) | 0.59 | |
| Norepinephrine requirement 24 h (n/%) | | | 158/63 | 118/79 | **<0.001** | |
| Norepinephrine requirement 48 h (n/%), *n=343* | | | 117/51 | 84/74 | **<0.001** | |
| Norepinephrine requirement 72 h (n/%), *n=305* | | | 78/37 | 67/71 | **<0.001** | |
| Norepinephrine dose (µg/kg/min) | | | 0.10 (0.01-0.19) | 0.15 (0.05-0.23) | **0.005** | |
| Maximum norepinephrine dose (µg/kg/min) | | | 0.19 (0.11-0.31) | 0.22 (0.17-0.40) | **<0.001** | |
| Mean arterial pressure (mmHg) | | | 71 (64-83) | 68 (60-79) | **0.02** | |
| Hourly diuresis at start of dialysis (ml/h) | | | 8 (0-48) | 3 (0-18) | **0.005** | |
| Mean hourly diuresis 0-24 h after start of dialysis (ml/h) | | | 10 (3-36) | 5 (1-20) | **0.002** | |
| Mean hourly diuresis 24-48 h after start of dialysis (ml/h) | | | 7 (2-25) | 3 (1-18) | **0.006** | |
| Mean hourly diuresis 48-72 h after start of dialysis (ml/h) | | | 7 (1-28) | 2 (1-21) | **0.007** | |
| Cumulative furosemide dose 0-72 h after start of dialysis (mg) | | | 10 (0-45) | 10 (0-40) | 0.18 | |
| pH | | | 7.33 (7.25-7.37) | 7.27 (7.19-7.34) | **<0.001** | |
| Bicarbonate (mmol/l) | | | 19.9 (16.8-21.8) | 16.8 (14.0-19.3) | **<0.001** | |
| Lactate (mmol/l) | | | 1.5 (1.0-3.3) | 3.4 (1.7-7.0) | **<0.001** | |
| Creatinine (µmol/l) | | | 331 (225-465) | 299 (208-411) | 0.20 | |
| Urea (mmol/l) | | | 20.2 (14.1-30.1) | 18.2 (12.4-25.0) | 0.13 | |
| Hemoglobin (g/l) | | | 101 (91-113) | 107 (92-112) | **0.03** | |
| Platelets (10^9^/l) | | | 143 (86-222) | 129 (70-208) | 0.08 | |
| International normalized ratio | | | 1.4 (1.2-1.9) | 1.5 (1.2-2.1) | 0.31 | |
| Bilirubin (µmol/l) | | | 12 (7-31) | 15 (9-32) | 0.14 | |
| Hospital mortality (n/%) | | | 62/25 | 72/48 | **<0.001** | |
| Duration of hospital stay, all patients (days) | | | 20 (10-33) | 13 (5-24) | **<0.001** | |
| Duration of hospital stay, hospital survivors (days) | | | 22 (12-35) | 20 (13-37) | 0.44 | |

Values are given at CRRT initiation, unless stated otherwise.

BMI = Body Mass Index; CRRT = Continuous Renal Replacement Therapy; SOFA = Sequential Organ Failure Assessment Score; SAPS-II = Simplified Acute Physiology Score; APACHE-II = Acute Physiology And Chronic Health Evaluation Score II

**Supplemental Table S2.** Univariate and multivariable associations between risk factors and hospital mortality

| **Variable** | **Univariate analysis** | | | **Multivariable analysis** | | |
| --- | --- | --- | --- | --- | --- | --- |
|  | **β** | **HR (95%CI)** | **P-value** | **β** | **HR (95%CI)** | **P-value** |
| Age (years) | 0.021 | 1.021 (1.006-1.036) | 0.005 | 0.033 | 1.033 (1.015-1.052) | 0.0003 |
| FB^net^ (ml/kg/h) | 0.424 | 1.529 (1.242-1.881) | <0.0001 | 0.302 | 1.353 (1.063-1.722) | 0.01 |
| Lactate (mmol/l) | 0.153 | 1.165 (1.126-1.205) | <0.0001 | 0.087 | 1.091 (1.034-1.151) | 0.002 |
| SOFA score | 0.122 | 1.130 (1.078-1.184) | <0.0001 | 0.068 | 1.070 (1.009-1.134) | 0.02 |
| Noradrenalin dose (0.1 µmol/kg/min) * | 0.219 | 1.245 (1.134-1.367) | <0.0001 | 0.069 | 1.071 (0.926-1.238) | 0.36 |
| FB^cum^ at CRRT initiation (litres) | 0.029 | 1.030 (0.995-1.066) | 0.09 | 0.032 | 1.032 (0.989- 1.077) | 0.15 |

FB^net^ = Hourly fluid balance per bodyweight during CRRT; SOFA = Sequential Organ Failure Assessment Score; FB^cum^ = Cumulative fluid balance.

*, Noradrenalin dose was excluded from the final multivariable model due to p=ns
